# Current Status of Neuromodulation-Induced Cortical Prehabilitation and Considerations for Treatment Pathways in Lower-Grade Glioma Surgery

**DOI:** 10.3390/life12040466

**Published:** 2022-03-22

**Authors:** Ryan P. Hamer, Tseng Tsai Yeo

**Affiliations:** 1 Faculty of Medicine & Health, University of Sydney, Sydney, NSW 2050, Australia; 2 Division of Neurosurgery, Department of General Surgery, National University Hospital, National University Health Systems, Singapore 119074, Singapore; tseng_tsai_yeo@nuhs.edu.sg

**Keywords:** neural plasticity, cortical prehabilitation, lower grade glioma surgery, awake brain mapping, navigated transcranial magnetic stimulation, direct cortical stimulation

## Abstract

The infiltrative character of supratentorial lower grade glioma makes it possible for eloquent neural pathways to remain within tumoural tissue, which renders complete surgical resection challenging. Neuromodulation-Induced Cortical Prehabilitation (NICP) is intended to reduce the likelihood of premeditated neurologic sequelae that otherwise would have resulted in extensive rehabilitation or permanent injury following surgery. This review aims to conceptualise current approaches involving Repetitive Transcranial Magnetic Stimulation (rTMS-NICP) and extraoperative Direct Cortical Stimulation (eDCS-NICP) for the purposes of inducing cortical reorganisation prior to surgery, with considerations derived from psychiatric, rehabilitative and electrophysiologic findings related to previous reports of prehabilitation. Despite the promise of reduced risk and incidence of neurologic injury in glioma surgery, the current data indicates a broad but compelling possibility of effective cortical prehabilitation relating to perisylvian cortex, though it remains an under-explored investigational tool. Preliminary findings may prove sufficient for the continued investigation of prehabilitation in small-volume lower-grade tumour or epilepsy patients. However, considering the very low number of peer-reviewed case reports, optimal stimulation parameters and duration of therapy necessary to catalyse functional reorganisation remain equivocal. The non-invasive nature and low risk profile of rTMS-NICP may permit larger sample sizes and control groups until such time that eDCS-NICP protocols can be further elucidated.

## 1. Introduction

Cerebral plasticity is considered an intrinsic property of the human nervous system, one that is continuously changing as a consequence of all neural activity. In the instance of pathophysiologic processes, intra- and inter-hemispheric interactions may shift over time to promote the establishment of new structural or functional changes [1]. The brain’s capability of reorganising itself in light of said processes has been particularly apparent in the clinical context of ischaemic stroke, whereby the sudden loss of specialized neural tissue may indicate a reorganisation of homologous functional architecture [2]. Similar mechanisms are suspected to be associated with slow-growing lower-grade supratentorial gliomas, often associated with focal, functional, connectional or connectomic diaschisis resulting in cortical inhibition or excitation, which in turn contributes to cerebral plasticity over time [3]. Confirmation of resulting ‘plastic’ mechanisms are often associated with metabolic changes and subsequent vascular coupling as indicated by functional magnetic resonance imaging (fMRI), or during surgery with Direct Cortical Stimulation (DCS), however, recent findings suggest that modalities such as navigated transcranial magnetic stimulation (nTMS) and magnetoencephalography (MEG) are also effective investigational tools capable of establishing analytical frameworks relating to connectomics and disease progression [4].

Historically, focal cortical diaschisis is only partially correlated with behavioural changes [3], contrary to classical or localisationist interpretations that have suggested otherwise. Localisationism refers to the notion that the brain is organised topologically, as originally indicated by Broca’s (1861) description of damage to the inferior frontal gyrus resulting in speech-related deficits and supported by Wernicke (1874) who associated the left superior posterior temporal gyrus with speech comprehension. Despite Brown-Sequard’s (1875) hypothesis that areas beyond the associative inferior frontal gyrus may also be linked with clinical presentation, localisationism remained at the forefront of scientific investigation relating to cerebral function for decades, the remnants of which are still apparent in neuroanatomical terminology today.

In contemporary clinical practice, it is presumed that cerebral function is organised hodotopically via a series of interconnected meta-networks involving both hemispheres responsible for various cognition, speech, motor, and sensory faculties [5]. Broca’s and Wernicke’s respective cortical areas are considered reductionist representations of eloquent language regions [6,7,8] but are still often used as referential landmarks. Contemporary models of speech and language processing are regarded as a dual-stream model [9,10], whereby the dorsal stream involves sensorimotor integration including the arcuate fasciculus and superior longitudinal fasciculus, and the ventral stream involves comprehension and includes the inferior frontal-occipital fasciculus, uncinate fasciculus and middle and inferior longitudinal fasciculi. Whereas it was once considered disastrous to risk damage to cortical structures comprising the classical Broca-Wernicke-Lichtheim-Geshwind model of speech function, it has since been widely contested, with data indicating that resection of these very structures does not contribute to permanent injury provided subcortical white matter tracts are not compromised [8,11,12,13,14,15,16].

Advances in functional neuro-imaging technologies such as task-based and resting-state functional Magnetic Resonance Imaging (rs-fMRI), MEG, nTMS, Diffusion Tensor Imaging Fiber Tracking (DTI-FT) and techniques relating to DCS have contributed to a greater comprehension of this connectomic theory during surgery for intra-axial brain tumours, which inadvertently provides an investigational landscape to uncover how the brain might be capable of reorganising eloquent functional networks with regard to iatrogenic mechanical or ischaemic injury, rehabilitation, disease progression and recently, concepts of cortical “prehabilitation”, which attempts to simulate a virtual lesion involving tumoural or peri-tumoural tissue to induce diaschisis and subsequent cerebral plasticity to avoid or reduce severity of premeditated post-operative deficits.

Task-based fMRI has become the dominant modality for functional brain imaging in both the clinical and the research community as a safe and painless clinical investigation tool. It allows for whole-brain coverage, including the ability to examine activity in deep brain structures. Assessment of neural function depends upon the Blood Oxygen Level Dependent (BOLD) signal, which is a surrogate marker of the changing ratios of oxyhemoglobin and deoxyhemoglobin in metabolically active brain regions. fMRI protocols for language assessment are not always reliable in comparison with DCS data [17], though the predictive value of motor regions is often sufficient [18]. In contrast, rs-fMRI measures spontaneous low-frequency fluctuations in BOLD signals with the patient at rest and without tasks, which is less demanding and can simultaneously identify many networks [19].

MEG measures diminutive magnetic fields created by bioelectric currents outside of the head that are generated by neural activity. In contrast to fMRI, MEG does not depend upon surrogate markers and offers excellent temporal resolution (<1 ms) because it directly measures these fields. MEG can be limited by its relative preference for specifically oriented field sources, as it primarily senses the tangential currents in the brain closer to the surface. Studies comparing pre-operative MEG with intraoperative DCS are compelling for motor regions [20,21] as well as language activation and lateralisation [22].

TMS applies a brief pulse of high-strength magnetic field over the scalp which passes through the skull and induces an electrical current in the underlying brain region [23], based on Faraday’s principle of electromagnetic induction. The current depolarizes a population of neurons, resulting in action potentials [24]. By altering the protocol of stimulation, TMS can cause either a temporary excitation or inhibition in the cortex. For neurosurgical and psychiatric applications, Navigated Transcranial Magnetic Stimulation (nTMS) incorporates a stereotactic system with the patient’s MRI designed to cater to the inter-individual variability of functional anatomy in addition to conformations of the human skull and cortex to provide accurate and precise magnetic stimulation and subsequent localisation of motor and speech areas of the brain [25,26]. nTMS has been shown to yield a close corroboration with DCS [27], however, divergent data has been reported for motor cortex localisation between nTMS and fMRI, ranging between 9.6 ± 7.9 mm for upper extremity and 15.0 ± 12.8 mm for lower extremity motor responses [28].

DTI-FT uses the direction of water diffusion parallel to white matter tracts as a surrogate marker for visualisation, which is able to graphically distinguish fiber bundles originating from a defined cortical or subcortical Region of Interest (ROI) often relating to the corticospinal tract or subcortical white matter pathways relating to speech and language relative to the tumour (for a review, Henderson et al. [29]). ROIs can be selectively defined based upon presumptive anatomic models of function, or integrated with functional data from fMRI, MEG or nTMS to reduce anatomic ambiguity associated with complex neoplasms such as glioma. DTI-FT data is incorporated with stereotactic navigation and used for pre-surgical planning and guidance during surgery.

This review aims to conceptualise the current state of invasive and non-invasive neuromodulation for the purposes of inducing cortical reorganisation, referred to as Neuromodulation-Induced Cortical Prehabilitation (NICP), to reduce the risk of premeditated post-operative neurologic sequelae following eloquent brain tumour surgery. We will first describe current surgical approaches and outcomes relating to lower grade glioma surgery. In section two, we explore electrophysiologic mechanisms and techniques relating to invasive and non-invasive brain mapping in glioma surgery. Section three details current applications of non-invasive neuromodulation in psychiatric and rehabilitation settings. Section four then relates previous sections to current case studies attempting to catalyse NICP in brain tumour patients harbouring perisylvian tumours. Section five then considers several neuroethical implications of prehabilitation relating to clinical decision making in lower grade glioma surgery, and whether or not current data supports the notion that current applications of NICP may permit future indications of reduced risk and improved post-operative course without transient injury and subsequent exhaustive rehabilitative therapy.

Focused, non-systematic literature searches were conducted to identify studies relating to sections one, two and three in consideration of section four and as a conceptual basis for section five. The authors nominated this approach in consideration of the vast undertaking required for systematic reviews for each section, however, a systematic literature search was reserved for studies relating to neuromodulation-induced cortical prehabilitation, of which no existing review protocol exists. The authors searched PubMed, Scopus and MEDLINE databases for articles published until December 2021 with the following MESH terms for section four: “cortical prehabilitation”, “functional reorganisation”, “neural plasticity”, “cerebral plasticity”, “eloquent brain tumour”. Inclusion criteria were studies investigating invasive or non-invasive neuromodulation to induce functional cortical reorganisation to reduce the risk of neurologic sequelae.

## 2. Current Surgical Approaches and Outcomes Relating to Lower Grade Glioma Surgery

The dilemma of brain tumour surgery involving the eloquent cortex is maximizing the extent of resection whilst minimizing neurologic morbidity, referred to as the onco-functional balance. Clinical approaches and philosophies are widely heterogeneous with regard to glioma surgery; however, it is universally accepted that safe and maximal resection with avoidance of significant ischaemic or neurologic sequelae is desirable. Part of the risk analyses to determine patient eligibility of brain tumour surgery often considers anatomical landmarks associated with eloquent function as indicated by proximity to classical (i.e., perirolandic or perisylvian) anatomic regions, or presumed functional cortex indicated by neuroimaging. Manipulation or resection near-to or within functional areas during surgery can result in transient or permanent neurologic injury, which can be severely debilitating in addition to leading to a poor prognosis. As such, careful consideration is made during surgery to reduce risk to these areas often with the integration of neuroimaging data and stereotactic navigation, with guidance from DCS.

Gliomas in particular can be located within close proximity or attached to these functionally important cortical areas and can also infiltrate subcortical white matter tracts. In a review by Krishna et al. [30], it has been posited that there are three levels of network reorganization relating to the development of lower grade glioma. First are the eloquent pathways intact within the tumour, second is the recruitment of pathways adjacent to the tumour, and third is the recruitment of ipsilateral or pre-existing contralateral connections. Glioma-related aphasia, for example, is thought to occur subject to infiltration at the cortical or subcortical level, iatrogenic injury during surgical intervention, or subsequent adjuvant radiation therapy. Early surgical intervention and gross-total or supratotal resection reduces the risk of infiltration and is well substantiated in prolonging overall survival in lower-grade gliomas [31,32,33,34] and higher-grade gliomas [35,36,37,38,39,40,41].

Lower grade gliomas (LGG; WHO grade I-II) grow slowly over time and are believed to induce cortical plasticity, with some recommendations that tumour resection be staged to preserve eloquence after observing functional reorganization between procedures [42]. The infiltrative character of lower grade glioma, however, makes it possible for functional components to remain within tumoural tissue, which renders complete resection without risk of neurologic deficit challenging. Although lower grade glioma patients have more favourable survival rates than patients with higher grade glioma (HGG; WHO grade III-IV), LGG is a uniformly fatal disease with survival averaging approximately seven to ten years [13] and reports of up to fifteen years [43], with all LGG’s systematically evolving towards higher grade anaplastic transformation and eventual demise [44].

Glioma surgery involving presumed functional cortex or subcortical white matter tracts is performed with the patient awake or asleep, though the criteria for either approach differs among neurosurgical centres. Asleep craniotomy under general anaesthesia is typically indicated for perirolandic regions unlikely to involve speech and language function. Awake craniotomy involves either conscious sedation or “asleep-awake-asleep” sedation and is classically reserved for perisylvian regions with the patient sedated but aware and responsive so that they can adequately perform neuropsychological speech assessment tasks during surgery aided with DCS.

Awake craniotomy with brain mapping is often clinically effective but technically demanding for both the patient and the surgeon. Considerations for surgery include medical comorbidities, neurological status, seizure frequency, body habitus and psychological status [45]. In preparation for awake surgery, a battery of neuropsychological examinations is conducted prior to and following surgery to assess the patient’s candidacy and whether or not there are deficits relating to cognition and speech function. Careful consideration must be made regarding pre-surgical neuroimaging studies to determine laterality or localisation of eloquent speech or motor regions, though these are sometimes considered insufficient to accurately exclude function and can be invalidated by intraoperative DCS findings.

During surgery, awake brain mapping tasks are individually tailored to assess suspected speech and language pathways affected or within proximity to the tumour or subcortical tracts, often including counting, comprehension, picture naming and semantic association, among others. DCS applied during these tasks attempts to disrupt neural pathways to confirm or disprove eloquent cortical or subcortical involvement subject to site of stimulation as indicated by the induction of aphasia, dysarthria, semantic paraphasia, amongst others, interpreted by neuropsychology or speech therapy teams and reported to the surgeon. Neuropsychological analysis during awake surgery is also heterogenous, with reports of high interrater variability, variability of selection and interpretation of intraoperative tasks, and subsequent indication of surgical termination points [46].

Awake craniotomy even without brain mapping is often still regarded as the ‘gold standard’ of functional assuredness during surgery as it provides ‘online’ feedback in real time without being affected by brain shift or anaesthesia. Outcome data also demonstrates that classically inoperable lesions involving eloquent cortex can be safely resected if aided by DCS, with most post-operative deficits reported to be mild (Medical Research Council grade 4+), or transient in nature [47,48]. Survival rates and neurologic outcomes do not necessarily differ between awake or asleep approaches [49,50,51].

Poor surgical outcomes have been independently predicted by advanced age, poor performance, pre-existing motor, language deficits [52] and tumour location [43,53]. Surgical goals are seldom standardised and are subject to tumour grading, progression of disease, and a mutually agreed upon treatment plan between patient and surgeon in consideration of post-operative QOL and adjuvant therapies. Complication rates vary in glioma surgery, however, there are numerous studies highlighting the reduction in neurologic morbidity in groups with DCS compared to those without [53,54].

Oncological outcome is typically measured as overall progression free survival or time to malignant transformation, whereas functional outcomes relate to the patient’s ability to communicate and move, though these assessments are not standardised and widely subjective [55]. Rates of permanent neurologic deficits range from 3 to 47% [45] and are sometimes reported to reach up to 66% [56]. Specific clinical circumstances may permit acceptance of a transient motor or speech injury during greater resection attempts, such as tumours involving the supplementary motor area (SMA) or classical speech related cortex, provided ischaemic or mechanical injury to underlying white matter tracts are avoided. SMA syndrome results in a transient hemiplegia lasting up to nine weeks [57], whereas restoration of normal language function following iatrogenic injury typically occurs within one to three months with extensive speech therapy [12].

Overall, transient neurologic injury following attempts of maximal resection in awake or asleep brain surgery is tolerated by multidisciplinary neurosurgical teams with the expertise and experience to conduct comprehensive brain mapping, integrate neuro-imaging data, and facilitate effective neurorehabilitation [58]. Current prospects of cortical prehabilitation entertain the possibility of avoiding these kinds of transient injuries altogether, derived from the investigational, diagnostic, and prognostic capabilities of clinical electrophysiology relating to brain tumour surgery.

## 3. Electrophysiologic Mechanisms and Techniques Relating to Invasive and Non-Invasive Brain Mapping in Glioma Surgery

Intraoperative brain mapping strategies are widely heterogeneous in practice. Various DCS configurations exist that are described in greater detail elsewhere [59,60,61,62,63,64,65]. Generally, Low-Frequency (LF) bipolar DCS is reserved for speech, language, and cognitive mapping. High Frequency (HF) monopolar DCS is usually reserved for motor mapping. Although there is recent data to suggest that a HF-DCS might also be practical for motor-speech mapping [66,67,68,69], a combination of both, and other, modalities are recommended amongst the literature, which can be used to mitigate the risk to eloquent cortex in the instance of increased anatomo-functional variability observed in supratentorial gliomas [70].

From an electrophysiologic perspective, bipolar LF-DCS provides a 50–60 Hz biphasic pulse with 1 ms pulse width and interstimulus interval of ~16 Hz, with intensity ranging typically between 0.5–20 mA. This is typically regarded as the Penfield technique [71], popularised by Ojemann [72,73,74], and is predominantly used as an inhibitory stimulus otherwise referred to as a “virtual lesion” capable of disrupting eloquent speech and language networks. In comparison, monopolar HF-DCS provides a 250–500 Hz monophasic train (usually between 2–8) with 0.5 ms pulse width and interstimulus interval of ~3 Hz, also ranging from 0.5–20 mA. Given the increased frequency and shorter stimulation, HF-DCS is particularly useful for cortico-subcortical motor mapping due to the effective recruitment of pyramidal cells and activation of the corticospinal tract. This technique is frequently used as an interventional tool for the detection of proximity to descending corticospinal tracts [75] as it can permit a more extensive surgical resection.

Although LF-DCS is also useful for mapping of motor regions, HF-DCS is preferred as it permits objective measurement during general anaesthesia and is often associated with a lower incidence of intraoperative seizure activity [76,77,78,79]. Either technique is subject to anaesthesia requirements, which differs amongst centres. Though it has also been reported that corticospinal tract proximity can be observed more frequently with HF-DCS under general anaesthesia in comparison to awake craniotomy without increasing the risk of mechanical related injury [76,79], LF-DCS remains a popular technique among neurosurgeons as it often negates the requirement for consulting clinical neurophysiology personnel demanded by HF-DCS, which incorporates other modalities such as Electrocorticography (ECoG).

Glioma surgery with brain mapping is associated with fewer late and severe neurologic deficits and a more extensive tumour resection, with recommendations to instate mapping as a standard of care [54]. There are a high volume of case reports exploring the clinical utility of DCS contributing to a reduction in neurologic morbidity involving motor and speech-related tumours [46,47,52,53,54,55,58,61,62,63,64,65,66,67,68,69,70,71,72,73,74,75,76,77,78,79,80,81], with recent reports of multilingualism [76,82,83,84], executive function [85,86,87,88,89], visuospatial attention [90,91,92] and working memory [93]. Paradigms have also been explored relating to higher order function such as singing [94], music performance [95], the integration of virtual reality-based tasks [96] and the prospect of cortico-cortical evoked potentials relating to speech function [97,98], which may permit asleep craniotomies for patients unsuited to awake surgery.

Fundamental principles of neurophysiological excitation and inhibition derived from DCS are similar in single-pulse nTMS and repetitive nTMS (rTMS), where low frequency (≤1 Hz) stimulation decreases cortical excitability and high frequency (≤5 Hz) stimulation increases cortical excitability. Pulses can be monophasic or biphasic in nature. Similarly, intermittent theta-burst rTMS (50 Hz) increases cortical excitability, whereas continuous theta-burst rTMS decreases cortical excitability. For greater detail, refer to position statements by Lefaucheuer et al. [99] and Oliviero et al. [100]. In short, an electrical current is discharged through a figure-eight TMS coil, generating a brief, cone-shaped magnetic field. This magnetic field can penetrate the skin, skull, and meninges without encountering significant distortion and induces an electrical field in the underlying brain [101]. TMS parameters are not universally applied, akin to brain mapping paradigms in neurosurgical applications that involve train count, pulse width, pulse duration, frequency and stimulation intensity (refer to He et al. [102] for a useful graphical summary of commonly used non-invasive brain stimulation protocols). Exact mechanisms of rTMS modulation are yet to be elucidated, and clinical results may vary. rTMS is believed to affect molecular pathways of the brain involving brain-derived neurotrophic factor (BDNF), dopamine, gamma aminobutyric acid (GABA), glutamate, serotonin, cortisol, or endogenous opioids [103].

nTMS is considered to be an emerging neuroimaging tool in glioma surgery. It has been reported to permit greater resection [104,105,106,107,108,109,110,111,112,113,114,115,116], increase progression-free survival in lower grade tumour patients when partnered with DCS [109], reduce craniotomy size [109,110], decrease operative time [108], improve surgical planning and designate clinically-useful regions of interest for DTI-FT [105,108], and reduce the time necessary for DCS mapping [109,111], which in turn reduces the risk of seizures during surgery. nTMS can also generate prognostic information regarding degree of disease infiltration [104,105,106,107,108,109,112,113,114] and has been shown to alleviate patient anxiety and improve patient comprehension regarding their procedure [105]. Recently, nTMS analysis following brain tumour surgery has provided prognostic utility relating to supplementary motor area syndrome or suspected motor injury [115].

There are various reports of a close correlation between nTMS and DCS data. Jeltema et al. [27] performed a systematic review comparing nTMS and DCS, which included 35 publications describing a total of 552 patients. For motor mapping, the distances between the cortical representation of the different muscle groups identified by nTMS and DCS varied between 2 mm and 16 mm. Most authors included in the study conclude that nTMS motor mapping is reliable in comparison to DCS mapping. Regarding nTMS speech mapping, the reviewers reported that sensitivity and specificity ranged from 10–100% and 13–98%, respectively, compared with DCS mapping. The positive predictive value (PPV) and negative predictive value (NPV) ranged from 17–75% and 57–100%, respectively. Most articles concluded that nTMS speech mapping was clinically useful, especially with regard to negatively mapped data within proximity to commonly eloquent cortical areas, meaning that those areas could be safely resected without impacting speech function.

Similarly, Raffa et al. [114] conducted a systematic review and meta-analysis with eight studies considered eligible. Results indicated that nTMS motor mapping significantly reduced the risk of postoperative new permanent motor deficits and increased the rate of gross total resection. The study concluded that preoperative nTMS mapping is associated with a reduced occurrence of postoperative permanent motor deficits, an increased gross total resection rate, and a tailored surgical approach compared to procedures without using preoperative nTMS mapping.

There is also recent data to suggest that a lesion to tract distance of <16 mm for the arcuate fasciculus and <25 mm for other close-by language tracts increases the risk of speech injuries post-operatively [113]. Similarly, for motor systems, <8 mm distance between tumour and the corticospinal tract, and an interhemispheric resting motor threshold of <90% or >110% is associated with a greater incidence of post-operative motor deficits [116,117], likely associated with altered motor excitability representative of significant disease infiltration [117,118].

These findings have important implications for surgical planning and pre-surgical risk stratification. It is generally accepted that lesions involving classical or topologic primary motor (periorolandic) and language (perisylvian) regions may necessitate either (a) an awake functional resection with DCS, (b) an awake functional resection without DCS, (c) an asleep anatomic resection with DCS. or (d) an asleep anatomic resection without DCS. nTMS is capable of identifying any risks to eloquent cortex prior to surgery to determine individually tailored surgical approaches, and there are also preliminary findings to suggest that this data may permit asleep surgery for patients that are ineligible for awake surgery [119].

With compelling spatial resolution for the accurate localisation of eloquent cortical topography, nTMS has also been used to map facial recognition [120], visuospatial attention [121,122,123], disorders of consciousness [124] and bilingual language localisation [125]. It has also proven to be a useful clinical tool for the non-invasive mapping of resulting plasticity following revision brain tumour surgery [126,127], which may present therapeutic opportunities relating to how the brain is capable of reorganising itself in combination with data generated via DCS. Other forms of non-invasive brain stimulation, such as transcranial Direct Current Stimulation (tDCS), may also provide therapeutic yield in LGG patients [128] but is similarly under-explored.

The wide heterogeneity of tumour progression patterns in addition to inter-individual variability of eloquent cortex and subsequent description of ‘meta-networks’ relating to cognitive function renders the standardisation of brain mapping and brain imaging challenging, which renders each procedure investigational and as such should be individually tailored. With the ultimate goal of preserving neurologic function with safe and maximal tumour resection, the information gathered during brain tumour surgery can also contribute to a comprehensive and potentially mechanistic understanding of network level activation relating to the hodotopic organisation of human brain function. As the wider literature continues to increase reports of brain mapping techniques used in the treatment of lower grade glioma, combined with improved neuro-imaging methodologies relating to the assessment of neural function, there has been increasing interest in commandeering the potential neuromodulatory effects of emerging clinical technologies to induce functional organization under circumstances where eloquent regions infiltrated by tumour are considered ‘inoperable’.

## 4. Current Applications of Non-Invasive Neuromodulation in Psychiatric and Rehabilitation Settings

In contrast to nTMS as a pre-surgical neuroimaging modality for functional localisation, rTMS is capable of modulating neural activity beyond the stimulation period. This is often related to notions of long-term potentiation (LTP), which enhances synaptic strength between regions, or long-term depression (LTD), which depresses synaptic activity. This has been comprehensively described [129] and has potentially therapeutic implications for neurological and psychiatric disorders attributed to mechanisms that are capable of modulating pre- and post-synaptic activity and, ultimately, changes in the excitability of cortical circuits that outlast the period of stimulation.

Therapeutic applications of non-invasive neuromodulation target neurologic biomarkers often indicated by fMRI and related to clinical diagnoses to ameliorate symptoms associated with the disorder under investigation. Thus far, rTMS applications are considered an investigational but effective therapeutic tool for medication-resistant depression [99,130], bipolar disorder [131], anxiety disorders [132], phantom pain [133], tinnitus [134,135], empathy [136], insomnia [137], bladder function [138], craving and dependence in substance abuse [139], tremor [140], complex regional pain syndrome [141], neuropathic pain [142,143,144], hemispatial neglect [145], epilepsy [146], obsessive compulsive disorder [147], symptoms relating to Alzheimer’s disease [148], symptoms relating to schizophrenia [149,150], Tourette syndrome [151], and symptoms related to Parkinson’s Disease [152], among others. Formal recommendations [99] support a clinical benefit for rTMS and the treatment of medication-resistant depression targeting the left dorsolateral prefrontal cortex, as well as neuropathic pain targeting the contralateral M1, which aims to produce an analgesic effect comparable to surgically implanted cortical stimulators. However, similar systematic reviews cast doubt regarding efficacy in depression patients [153] and there are claims that significant benefits might be associated with a more reliable targeting of cortical regions [154], which reinforces the importance of standardised guidelines for research and practice.

Commonly targeted regions for psychiatric applications of rTMS include the anterior and lateral aspects of the dorsolateral prefrontal cortex (DLPFC) and associative networks, however, the frontopolar cortex, ventromedial prefrontal cortex and ventrolateral prefrontal cortex have also been associated with depression pathophysiology. In a systematic review exploring targeted biomarkers for rTMS therapy [103], depression was associated with increased GABA and BDNF levels, chronic pain was associated with increased levels of b-endorphin, and post-stroke neurologic deficit associated with decreased BDNF levels.

TMS is considered non-invasive thanks in part to its safety profile. When the parameters of stimulation are maintained within established ranges, the rates of complications associated with TMS protocols are low. Minor adverse effects include headache [155], temporary high-frequency hearing loss [156], and pain [155,156,157,158,159]. The most severe acute adverse effect is seizure. High frequency (“multipulse”) rTMS protocols have the greatest risk of precipitating seizure activity, whereas low-frequency trains have also been reported to cause seizure but at rates of less than 1% [157,158,159,160,161]. With regard to lower grade glioma patients, single-pulse nTMS protocols require exclusion of patients with ‘uncontrolled’ or ‘poorly controlled’ seizures, which are defined as a seizure frequency greater than one per week. These measures are intended to minimize the probability of provoking a seizure during nTMS mapping, however, large-cohort data suggests a strong safety profile in brain tumour patients [162] and epilepsy patients [161,162,163,164,165,166,167,168].

Beyond psychiatric applications, rTMS findings in stroke studies have contributed to a wider understanding of mechanisms involved in neuroplasticity, whereby the sudden loss of specialized neural tissue initiates a reorganisation of functional architecture. During the acute phase, damage in critical areas relating to motor or language function can contribute to global network dysfunction made worse if involving white matter tract disconnection [169]. In the subacute phase compensatory mechanisms of recovery become apparent via the recruitment of homologous areas. The primary factors hindering post-stroke functional recovery are synaptic function changes, such as decreased excitability of the affected hemisphere, and interhemispheric imbalance of inhibition caused by diaschisis. rTMS is thought to promote functional recovery by inducing the endogenous repair and recovery mechanisms of the brain [170] via activation of BDNF processing [171]. Compelling data has been reported on rTMS rehabilitation for stroke-induced motor and speech injury [172,173,174] and is fast becoming recognised as a viable therapeutic option [175,176,177,178,179]. Recent data also indicates that similar rTMS mechanisms may expedite motor recovery following iatrogenic surgical injury [180].

**Figure 1:**
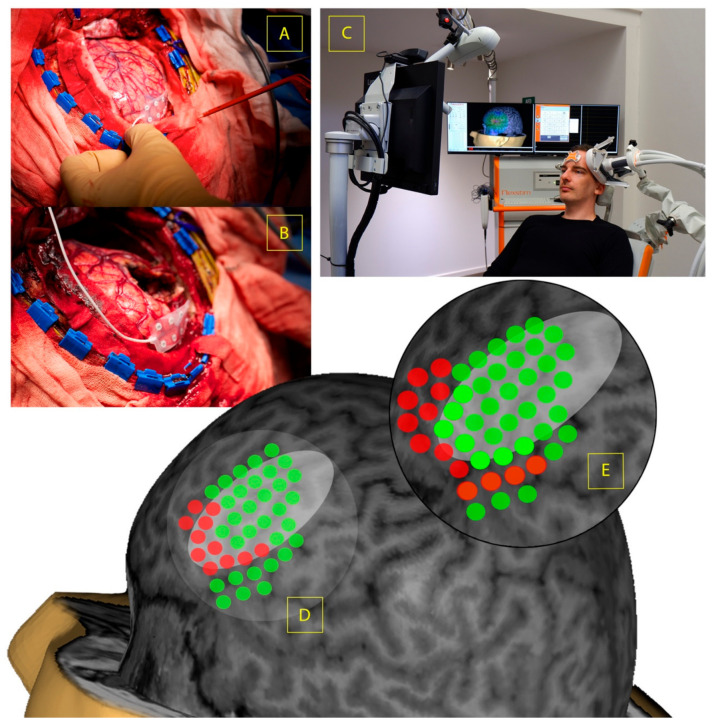
Illustrative example of neuromodulation-induced cortical prehabilitation. (**A**) Placement of subdural electrode to confirm eloquent cortical topography prior to surgical debulking. (**B**) End of tumour debulking as distinguished by definition of electrophysiologic or anatomic boundaries, subdural electrode remains for eDCS-NICP towards the pars opercularis/pars triangularis indicated by pre-operative imaging and brain mapping during part-A. (**C**) rTMS-NICP setup targeting the same cortical region as a non-invasive alternative to extraoperative cortical stimulation. (**D**) Schematic diagram indicating positive speech related areas (red), and negative areas (green) relating to the lesion (grey oval) prior to NICP. (**E**) Schematic diagram indicating confirmation of functional reorganisation following NICP as measured by neuroimaging, TMS and/or DCS.

Positive clinical outcomes in lower grade glioma surgery are frequently reported, though the risk of transient or permanent neurologic injury persists. It is evident that the integration of neuroimaging and brain mapping data is capable of investigating processes related to adaptive neural plasticity, however, data derived from psychiatric and rehabilitation studies indicate that cerebral function and related neural networks might well be harnessed for therapeutic benefit [181,182]. Recently, several groups have investigated the possibility of inducing neural plasticity in patients harbouring perisylvian lesions using non-invasive neuromodulation such as rTMS [183], or extraoperative DCS (eDCS) [184,185,186], henceforth referred to as neuromodulation-induced cortical prehabilitation (rTMS-NICP, eDCS-NICP; Figure 1). In addition to the potentially interventional and therapeutic benefit for patients, these approaches may be capable of providing meaningful insight regarding the voluntary manipulation of neural networks in order to permit safe surgical procedures or expedite recovery from surgical injury, however, these have not yet been extensively or systematically explored.

## 5. Current Case Studies Attempting to Induce Cortical Plasticity in Brain Tumour Patients

Similarly to neurophysiological mechanisms of deep brain stimulation, cortical reorganization initiated by neuromodulation-induced prehabilitation is still are matter of debate and has been far less investigated in comparison. The rationale is to regularly electrically modulate targeted functional regions or networks involving the tumour partnered with relevant neuropsychological or motor function tasks in order to catalyse cortical plasticity [184]. To complicate matters, there is no universal consensus regarding the definition of cortical plasticity. Prehabilitation in the context of this review relates to functional reorganization and subsequent objective measurement following invasive or non-invasive neuromodulation to permit safer surgical intervention and reduce the likelihood of premeditated neurologic injury relating to speech and language function. Several studies to date have explored the feasibility of rTMS-NICP and eDCS-NICP prior to revision glioma surgery (Table 1).

Barcia et al. [183] reported on their experience with a 59 y/o female undergoing awake resection of an oligodendroglioma involving pre- and postcentral sulci after presenting with dysnomia. Preoperative fMRI indicated suspected motor speech involvement and adjacent to Broca’s, with language activation also localised to the right hemisphere. Preoperative MEG data indicated left motor speech dominance. Her initial procedure was abandoned following detection of a nomination defect and loss of verbal function during DCS within the tumour cavity indicating involvement of the arcuate fasciculus. The extent of the initial resection was not specified, and the patient was subsequently treated with radiotherapy and temozolomide. Six months later she presented with dysarthria and dysphasia, with neuroimaging indicating infiltration of the inferior aspects of precentral and postcentral gyri and the posterior aspect of the inferior frontal gyrus. Theta-burst rTMS-NICP was targeted 1 cm anterior to motor cortex, which contributed to a difficulty in phonological fluency and articulation of words during stimulation. rTMS to this region was initiated daily for two weeks and three days, combined with speech therapy tasks derived from the Boston Diagnostic Aphasia Examination (BDAE). Revision surgery was completed nine months following her original procedure. Post-prehab MEG indicated greater cortical bilateralisation, whereas fMRI showed no discernible change. During revision surgery, frozen section histopathological analysis confirmed radiation necrosis. Post-operatively the patient experienced a transient deficit, eventually returning to baseline dysnomia symptoms. The authors concluded that the resulting MEG bilateralisation may not have been attributed to speech therapy alone.

In a separate case study, Barcia and colleagues [185] attempted eDCS-NICP in a 27 y/o male with an anaplastic astrocytoma infiltrating Broca’s area. The patient underwent a planned resection five years prior, which consequently resulted in a biopsy due to the detection of language pathways within tumour as indicated by pre-operative fMRI and confirmed intraoperatively by DCS. To initiate NICP for revision surgery, the surgical team performed an awake craniotomy and confirmed eloquent speech regions adjacent or involving the tumour, with subsequent implantation of a subdural grid electrode targeting these regions. Sequential testing of the electrode array post-operatively confirmed targeted channels for high-frequency stimulation extraoperatively (130 Hz and 1 ms fixed pulse width) as indicated by disruption of speech. Incremental stimulation was delivered continuously for twenty-five days and partnered with speech therapy. Two weeks following the implantation, subdural channels were sequentially tested and revealed a disparity in channels originally associated speech disturbance prior to the extraoperative testing period. Following surgery, the patient experienced mild dysarthria that swiftly recovered. Post-operative fMRI revealed new language activation at dominant and homologous frontal regions, with neuropsychological assessment indicating mild improvement in attention and speech function.

In a similar exploration of eDCS-NICP, Rivera-Rivera et al. [184] reported on 5 patients harbouring gliomas relating to Broca’s area (*n* = 4) and pre-central gyrus (*n* = 1), with a mean extraoperative mapping time of 18.8 days (130 Hz, 1 ms pulse width), and subsequent re-operation mean 33.6 days later. Extraoperative stimulation continued until it no longer resulted in functional deficits at maximal stimulation intensity. In contrast to Barcia et al.’s non-invasive approach [183], complication rates were high, with two patients experiencing deterioration of language function long-term, three patients experiencing focal seizures, and two with infection associated with craniotomy and electrode implantation. Total resection was not achieved in any procedure, with the authors observing that stimulative disruption was applied only for tumoural but not peri-tumoural areas, which may have reduced efficacy. Despite these results, the authors claim NICP was successful as indicated by fMRI and implanted cortical electrode array changes. In consideration of the high complication rate, the authors suggest that non-invasive stimulation procedures should also be explored.

Recently, Serrano-Castro et al. [186] investigated eDCS-NICP in a 17 y/o male originally diagnosed with a neuroepithelial dysembryoblastic tumour in the left temporo-parietal region resulting in refractory focal motor seizures. The patient originally underwent conservative resection as a 6 y/o to avoid language deficit, but subsequent functional investigation at 17 y/o revealed that the lesion was infiltrating Wernicke’s area. The patient underwent an awake craniotomy to confirm language regions related to tumoural and peritumoural tissue, with eDCS-NICP parameters akin to those aforementioned (130 Hz, 1 ms pulse width, intensity up to 10 V). This was the first report of prehabilitation in epilepsy surgery rather than malignant brain tumour surgery. The authors concluded eDCS-NICP contributed to a depression of activity relating to the tumour, and increased activation in dominant and homologous Wernicke’s area.

## 6. Technical and Neuroethical Implications of Prehabilitation Relating to Clinical Decision Making in Lower Grade Glioma Surgery

By definition, neuromodulation-induced cortical prehabilitation (NICP) is designed to prevent premeditated neurologic sequelae that otherwise would have resulted in extensive rehabilitation or permanent injury following surgery. The possibility of inducing functional reorganization by means of NICP in lower grade glioma or epilepsy patients may be feasible given the slow-growing nature of WHO grade I and II lesions. However, neuro-oncological strategies for the management of lower grade glioma favours early surgical intervention as the primary treatment option to facilitate opportunities of progression-free survival. Indeed, under circumstances where eloquent pathways are detected intraoperatively, it has been reported that surgical procedures can be staged and accompanied with speech and physiotherapy to initiate natural functional reorganisation [42]. Preliminary cortical prehabilitation findings represent the possibility that this may be avoided if proven to be effective, which may inevitably reduce the risk and demand for resources associated with surgery and rehabilitation.

There are inherent ethical considerations relating to the time necessary to catalyse cortical prehabilitation versus the earliest opportunity for surgical intervention to increase opportunities of progression-free survival. On average, WHO II tumour growth is reported to be approximately 4 mm per year [13]. Duration of rTMS-NICP and eDCS-NICP therapy ranged between six and twenty-five days in the aforementioned case studies, though the majority of patients in these reports still underwent some degree of rehabilitation following surgery. It is understood that the number of sessions is an important parameter to predict clinical efficacy [187]. Average rTMS treatment duration in patients with depression lasts approximately two weeks, with additional treatment beyond this period potentially resulting in improved clinical benefit. Similarly, optimal stimulation ‘dosage’ in stroke rehabilitation patients is often personalised in accordance with the time post-stroke and lesion site, resulting in modified number of pulses or length of therapy rather than increased stimulation intensity, with sessions up to sixteen days [188]. In the current series, the common feature representing facilitation of functional reorganization was indicated by negative speech disturbance at maximal stimulation intensity and subsequent functional bilateralisation indicated by fMRI or MEG, yet there was still a high incidence of post-surgical deficits, albeit transient.

Future prehabilitation studies may require longer periods of therapy than currently reported, however, if they prove to be fruitful, there are also resource and economic considerations relating to pre-operative prehabilitation pathways in comparison to the possibility of post-operative rehabilitation pathways. The aforementioned preliminary findings may prove sufficient for the continued investigation of prehabilitation in small-volume lower-grade tumour or epilepsy (i.e., focal cortical dysplasia) patients. However, in consideration of the very low number of peer-reviewed case reports it remains equivocal as to the stimulation ‘dosage’ parameters and duration necessary to facilitate NICP therapy.

It is understood that brain stimulation of any description can induce differential effects. Andoh et al. [189] noted that LF-rTMS was capable of facilitating detection of native languages, whereas theta-burst facilitated detection of foreign languages. Further, HF-rTMS (5–10 Hz) over Broca’s area has been associated with the facilitation of phonological and syntactic performance as well as impaired semantic performance, whereas LF-rTMS over Wernicke’s area has been shown to have no effect during picture naming tasks. Although the mechanism of action of rTMS speech mapping is not adequately elucidated, synchronization of affected neurons and GABAergic inhibition are thought to contribute to the temporary brain disruption and lesion effect [25]. Cortical prehabilitation therapy may require longer periods of therapy to initiate LTD associated with GABAergic inhibition in comparison to aforementioned stroke rehabilitation resulting in BDNF activation, or depression therapy targeting GABA and/or BDNF activation [103].

Interestingly, there are no further reports of rTMS-NICP following Barcia et al.’s description [183], which was theoretically sound in the context of previous applications in psychiatry and rehabilitation. The pattern of theta-burst rTMS is based on the brain’s natural theta rhythm occurring in the hippocampus and has also been used in animal studies to induce synaptic plasticity [190]. The duration of theta-burst rTMS after-effects is also subject to the pattern of stimulation. Intermittent stimulation (applied for 2 s and repeated every 10 s) targeting the motor cortex results in increased cortical excitability, whereas continuous stimulation (applied for 40 s without pause) results in depression of excitability. Data derived from theta-burst protocols appear to be more consistent than standard rTMS protocols, perhaps because intensity and number of pulses are approximately equal. This feature could be advantageous for the design of future studies exploring cortical prehabilitation.

In comparison to Barcia et al.’s [183] non-invasive approach, the common stimulation frequency for extraoperative direct cortical stimulation in this series was frequently reported to be 130 Hz with a 1 ms pulse width. This is purportedly nominated as they are similar to those used in deep brain stimulation (DBS), which are believed to inhibit cell bodies and reduce their firing rate while exciting axons and increasing their action potential frequency, resulting in therapeutic benefit. In DBS, however, the pulse width is often modified (60–400 us) in addition to stimulation intensity [191], and stimulation polarity [192]. Jakobs et al. [193] note that the electrical effect of DBS electrodes within the subthalamic nucleus are strongly influenced by the anisotropic nature of the tissue at the targeted site in relation to the position of the electrode, which can result in heterogenous therapeutic effect. The clinical standard of 130 Hz purportedly acts upon large, myelinated axons that then depolarise and result in action potentials, which then trigger neurotransmitter release once the stimulation is approximately twice as high as their intrinsic firing frequency. Long-term open-loop DBS has also shown to exhibit neuroplasticity even after deactivation of the device [192], promoting the development of closed-loop systems built upon rheobase and chronaxie analysis and subsequently resulting in interleaving stimulation and improved clinical results [193]. Further investigation regarding optimal stimulation parameters for prehabilitation should employ a similar type of analysis to ensure that results are clinically effective, akin to investigations originally pertaining to HF-DCS [194], to ensure that repetitive sessions of rTMS-NICP or eDCS-NICP avoid instances of tolerance or habituation effects associated with invariant stimulation. More importantly, this data would help establish the necessary criteria to permit the confirmation of adequate functional reorganisation and subsequent discontinuation of NICP therapy to proceed with surgery.

With preliminary findings suggesting that invasive or non-invasive neuromodulation may be achievable for inducing functional reorganization of perisylvian cortex or speech related networks, clinicians and tumour boards must consider the feasibility of inducing plasticity prior to surgery, delaying surgery after intraoperative detection of eloquent cortical pathways [42], or accepting a deficit with the risk of insufficient recovery. The potential for functional reorganization is not equal, however. Cortical regions such as the primary motor cortex are less ‘plastic’ in comparison to higher-order cortical networks such as the angular gyrus. Regions with less ‘concrete’ functional determination are thought to be associated with network-level activation, which renders reorganization possible. For example, supplementary motor area and the middle and inferior frontal gyri represent an intermediate level of functional integration [195], and if resected, results in an immediate but recoverable deficit. Factors relating to effective redistribution of function are linked to age, growth kinetics of the lesion, tumour location, affected function and gender [195]. As such, assumptions should not be made regarding effective redistribution of function, which may result in unwanted pathological consequences of maladaptive plasticity.

Functional reorganization must also be objectively measured and validated, ideally with predictive markers indicative of a clinical response, or lack thereof. Prospective research protocols would likely need to incorporate intrasubject evaluations of plasticity at various time points, ideally in comparison with healthy controls to highlight true signal variation reflective of functional changes that could be attributed to neuromodulation. With regard to fMRI, rs-fMRI and MEG, this would likely become a resource burden. However, for less-invasive modalities such as nTMS and Electroencephalography (EEG), or indeed an integrated nTMS-EEG platform, may prove more feasible for detection and progressive activation of homologous regions or modulation of GABAergic processes during therapy. Further, progressive nTMS or fMRI imaging during eDCS-NICP attempts may be marred by the influence of artifact associated with platinum iridium electrode coil arrays, which may be contraindicated or result in significant signal distortion. This may render rTMS-NICP as preferential during subsequent investigations, as nTMS has been reported to reliably measure cortical plasticity [126,195,196,197] and function-specific connectomes [198] in glioma patients. It is undisputed that the confirmation of successful cortical prehabilitation is likely best accomplished in corroboration with DCS findings during awake craniotomy and post-operative neurologic status, which will validate or invalidate the therapeutic efficacy of this approach as a viable treatment pathway.

Overall, a number of methodological considerations can be made from these studies to promote further investigation into the possibility of neuromodulation-induced prehabilitation. For one, a general consensus on the definition of cortical plasticity is yet to be considered uniform relative to lower grade glioma. This often refers to the complex and multifaceted mechanisms of the brain to compensate for impaired function and substantiated by the clinical investigation of stroke, epilepsy, and brain tumours, in addition to neuroimaging and psychiatric studies. Concepts of NICP should refer to the measurable induction or precipitation of functional reorganization, either invasively or non-invasively, that attempts to catalyse impaired function resulting in diaschisis and thus inducing plasticity. This can only be accomplished with a multidisciplinary clinical team.

Secondly, there are divergent methods of analysing functional changes from neuroimaging data and neurocognitive assessment. Despite the compelling and often sophisticated data provided by DCS, nTMS, rTMS, MEG, and fMRI, there are methodological limitations and significant variability not only in the application of these technologies, but also among patients. Future protocol design might consider a systematic approach to their investigations that could include homogenous subgroups relating to invasive versus non-invasive neuromodulation, suspected histopathology of tumour, anatomic location etc to ensure that data can be reliably associated with the successful or unsuccessful induction of diaschisis. Similarly, as a novel investigational protocol, it is important to maintain the ethical requirements that patients are provided informed consent, the benefit of the research outweighs the risks, and if applicable, there is an equal distribution of burdens and benefits across patients. Whilst evidence-based medicine calls for randomized controlled trials, in the current instance this remains a significant challenge given the significant heterogeneity of disease progression, neurologic status, neuropsychological status, psychosocial faculties, in addition to variable surgical strategies employed across neurosurgical centres globally. Until such trials can be responsibly designed and executed, prospective case series such as those aforementioned remain a valuable clinical and research opportunity.

## 7. Conclusions

The possibility of inducing cortical functional reorganization is definitely promising for lower grade gliomas in order to ensure a reduced likelihood of iatrogenic insult, which results in debilitating compromise of quality of life in addition to a poor prognosis. At the time of writing, early stage invasive or non-invasive prehabilitation findings are at best an experimental indication of utility that warrants continued and systematic investigation. The small series of reported case studies should be taken into consideration for larger cohorts and where possible compared with healthy controls. Despite the promise of such science fiction-like clinical intervention, there are inherent technical and neuroethical considerations to be made. It is evident that future studies exploring invasive or non-invasive neuromodulation in lower grade glioma or epilepsy patients will demand careful and highly selective patient recruitment, in order to avoid increased risks associated with disease progression. Given the non-invasive nature and low risk profile of rTMS-NICP, larger sample sizes are likely more achievable using this modality until such time that eDCS-NICP protocols are further elucidated.

It is unmistakeable that brain tumour patients have inadvertently provided a circumstance permissible to the wider understanding of neurocognitive function, often at the expense of an unfortunate prognosis. As doctors, surgeons, and scientists are gradually beginning to understand how the brain might be organised, and under which circumstances it is capable of reorganising itself, treatment paradigms in neuro-oncological surgery are evolving with the information gathered in its wake. Recent advances in functional neuro-imaging technology are capable of characterising the mechanisms of neural redistribution initiated by lower grade gliomas, and ultimately how the brain is capable of functional reorganisation during disease progression and following surgical resection. Whilst the current findings indicate a broad possibility of successful cortical prehabilitation relating to perisylvian cortex, invasive and non-invasive brain stimulation paradigms remain a compelling but under-explored investigational tool for the modulation of the eloquent brain to permit greater resection and reduce neurologic sequelae in lower grade glioma patients. Future studies should define patient selection criteria, optimal stimulation ‘dosage’ parameters, and duration of treatment, in addition to functional biomarkers for therapy discontinuation beyond the measurement of focal diaschisis, to establish whether cortical prehabilitation may represent a viable therapeutic pathway in contrast to the possibility of rehabilitation following surgery.

## Figures and Tables

**Table 1:** Summary of rTMS-NICP and eDCS-NICP case studies.

	Barcia et al. [183]	Barcia et al. [185]	Rivera-Rivera et al. [184]				Serrano-Castro [186]
Patient (s)	59 y/o F	27 y/o M	52 y/o F	34 y/o F	51 y/o M	41 y/o M	17 y/o M
Tumour	Oligodendroglioma (WHO II)	Anaplastic astrocytoma (WHO III)	Oligodendroglioma (WHO II)	Anaplastic oligodendroglioma (WHO III)	Anaplastic astrocytoma (WHO III)	Oligodendroglioma (WHO II)	Neuroepithelial dysembryoblastic tumour (WHO I)
Anatomy	Adjacent to left IFG	Left IFG	Left IFG, MFG, SFG	Left STG, MTG, ITG	Left PrCG	Left IFG, MFG, SFG	Left temporoparietal region
Presenting symptoms	Dysnomia	Speech impairment	Language function	Language production and function	Movement of right hand and shoulder	Movement of hand, language production	Focal motor seizures (right lower limb), aphasia without awareness
Pre-op imaging	fMRI: left dominant speech with partial right-side activation	fMRI: left dominant speech with activation within tumour	fMRI: left dominant bilingual speech	fMRI: left dominant speech	fMRI: right hand activation within tumour	fMRI: left dominant speech and motor function within tumour	fMRI: Overlap of Wernicke’s area and tumour, language reorganisation in homologous contralateral hemisphere
	MEG: left dominant speech						
Revision surgery (Y/N)	Y; initial 0.9 yrs prior	Y; initial 4.8 yrs prior	Y; initial 6.2 years prior	Y; initial 4.7 years prior	N	Y; initial 7.8 years prior	Y; initial approx. 11 years prior
Technique	Theta-burst rTMS	Extraoperative direct cortical stimulation					
*Power/Intensity*	60%	0.5–10 V (incremental)					
*Frequency*	45 Hz	130 Hz					
*Pulses*	3	200					
*Bursts/pulse width (ms)*	5	1 ms					
*Cycle duration (s)*	1 s	Continuous, 24 h p/day					
*Number of cycles*	40	n/a					
*Burst frequency (Hz)*	5	n/a					
Cognitive assessment	Boston Diagnostic Aphasia Examination (BDAE)	Mini-mental State examination, Boston Diagnostic Aphasia Examination, Token test, F-A-S Test (subset of Neurosensory Center Comprehensive Examination for Aphasia)	Object naming, repetition, pseudowords and phrases, understanding simple and complex orders, verbal fluency	Object naming, repetition, pseudowords and phrases, understanding simple and complex orders, verbal fluency	Right shoulder movements (elevation, abduction, and flexion), right elbow movements (flexion, extension, pronation, and supination), and right-hand fine motor movements (finger tapping, flexion and extension, abduction and adduction)	object naming, repetition, pseudowords and phrases, understanding simple and complex orders, verbal fluency. Right shoulder movements (elevation, abduction, and flexion), right elbow movements (flexion, extension, pronation, and supination), and right-hand fine motor movements (finger tapping, flexion and extension, abduction and adduction)	Boston Diagnostic Aphasia Examination (BDAE)
Length of Prehab (days)	13	25	16	16	22	15	6
Post-prehab imaging	fMRI: left dominant speech with partial right-side activation	fMRI: new language activation at ipsilateral and contralateral hemisphere	fMRI: reorganization of languages at basal aspect of left inferior gyrus	fMRI: new language activation at contralateral hemisphere	fMRI: displacement of motor function to the depth of the central sulcus	fMRI: reorganization of language and motor hand area	fMRI: decreased activation in left dominant hemisphere, greater activation in right homologous area of Wernicke’s
	MEG: greater bilateralization	Electrode array: disparity from original mapping	Electrode array: all contacts negative for Spanish, Romanian still present	Electrode array: 9 contacts originally provoking dysnomia and alexia no longer did so	Electrode array: all sites originally positive for motor activation were negative	Electrode array: 9/11 sites originally producing speech disturbances were negative, remaining pair producing phonological aphasia	Electrode array: no residual language over tumour region
Prehab complications	Nil	Focal seizures, osteomylitis of bone flap	Nil	Epidural abscess associated with worsening neurology	Intermittent myoclonus right index finger	Pre-prehab subdural hematoma, subsequent removal and re-implantation of subdural electrode	Nil
Neurologic status following surgery	Transient language deficit. BDAE lower than pre-surgery.	Transient dysarthria. Attention and speech function (BDAE) improved.	No new or worsening neurologic deficit	Long term language deterioration	Transient shoulder elevation difficulty	Slight motor aphasia, long term language deterioration	Nil

Abbreviations: M: male, F: female, WHO I: World Health Organisation tumour grade I, WHO II: World Health Organisation tumour grade II, WHO III: World Health Organisation tumour grade III, IFG: inferior frontal gyrus, MFG: middle frontal gyrus, SFG: superior frontal gyrus, STG: superior temporal gyrus, MTG: middle temporal gyrus, ITG: inferior temporal gyrus, PrCG: pre-central gyrus, fMRI: functional magnetic resonance imaging, MEG: magnetoencephalography, n/a: not applicable, nil: no data that meets criteria.

## Data Availability

Not applicable.
